# A global database of land management, land-use change and climate change effects on soil organic carbon

**DOI:** 10.1038/s41597-022-01318-1

**Published:** 2022-05-24

**Authors:** Damien Beillouin, Julien Demenois, Rémi Cardinael, David Berre, Marc Corbeels, Abigail Fallot, Annie Boyer, Frédéric Feder

**Affiliations:** 1 CIRAD, UPR HortSys, F-34398 Montpellier, France; 2 HortSys, Univ Montpellier, CIRAD, Montpellier, France; 3 CIRAD, UPR AIDA, F-34398 Montpellier, France; 4 AIDA, Univ Montpellier, CIRAD, Montpellier, France; 5 CIRAD, UPR AIDA, Harare, Zimbabwe; 6 Department of Plant Production Sciences and Technologies, University of Zimbabwe, Harare, Zimbabwe; 7 CIRAD, UPR AIDA, Bobo-Dioulasso, Burkina Faso; 8 CIRDES, USPAE, Bobo-Dioulasso, Burkina Faso; 9 IITA, Nairobi, Kenya; 10 CIRAD, UMR SENS, Montpellier, France; 11 SENS, Univ Montpellier, CIRAD, Montpellier, France; 12 CIRAD, DGDRS, DIST, F-34398 Montpellier, France; 13 CIRAD, UPR Recyclage et Risque, Montpellier, France; 14 Recyclage et Risque, Univ Montpellier, CIRAD, Montpellier, France

**Keywords:** Climate-change mitigation, Environmental impact

## Abstract

Increasing soil organic carbon (SOC) in natural and cultivated ecosystems is proposed as a natural climate solution to limit global warming. SOC dynamics is driven by numerous factors such as  land-use change, land management and climate change. The amount of additional carbon potentially stored in the soil is the subject of much debate in the scientific community. We present a global database compiling the results of 217 meta-analyses analyzing the effects of land management, land-use change and climate change on SOC. We report a total of 15,857 effect sizes, 6,550 directly related to soil carbon, and 9,307 related to other associated soil or plant variables. The database further synthesizes results of 13,632 unique primary studies across more than 150 countries that were used in the meta-analyses. Meta-analyses and their effect sizes and were classified by type of intervention and land use, outcomes, country and region. This database helps to understand the drivers of SOC sequestration, the associated co-benefits and potential drawbacks, and is a useful tool to guide future global climate change policies.

## Abstract (summary)

**Table:** 

Measurement(s)	soil organic carbon
Technology Type(s)	systematic literature review
Factor Type(s)	land-use change •land management • climate change
Sample Characteristic - Environment	anthropogenic environment • natural environment
Sample Characteristic - Location	global

## Background & Summary

Limiting the global mean temperature increase below 2 °C above mid-19th century levels would significantly reduce the risks and impacts of climate change^1,2^. Soil is a key compartment for climate regulation either by emitting greenhouse gases (GHGs) or by sequestering organic carbon (OC)^3^. The first meters of soils contain between 1,500 and 2,400 Pg OC^4–7^, *i.e.* three to four times the amount of carbon (C) present in the vegetation (450–650 Pg C) and two to three times the amount of C in the atmosphere (~829 Gt C).

Soil organic carbon (SOC) storage represents about 25% of the potential of natural climate solutions^8^ to offset global anthropogenic GHG emissions. Increasing SOC is also expected to provide other indirect benefits such as an improved capacity of soils to contribute to agricultural adaptation to climate change, and to enhanced food security^9–11^. These multiple benefits are the rationale of the “4 per 1000 Initiative: Soils for Food Security and Climate” (www.4p1000.org), which sets a global aspirational goal to increase SOC stocks in agricultural soils at an annual rate of 0.4% to a depth of 0.3–0.4 m. Yet, the achievability of such annual increase in agricultural SOC stocks has been intensively discussed and criticized. Biophysical limits (*e.g*. in terms of supply of water and nutrients) and other barriers such as the socio-economic implications for the agricultural sector, were raised by several authors^12–17^. The many biophysical factors affecting SOC storage (*e.g.* biomass and nutrient input levels, land-use change, pedoclimatic conditions, and climate change) and their spatial and temporal variability and interactions confound a clear comprehension of their effects on SOC storage despite the exponential increase in the number of studies on SOC. The evidence is fragmented^18^, too often characterized by studies of variable quality- and by poor analyses of trade-offs with other variables (e.g. nutrient availability^19–21^). As a consequence, strategies to scale out management options for increased SOC storage are still debated^22^.

Our database provides a worldwide exhaustive and quantitative assessment of available meta-analyses about the impact of major interventions on SOC. It contains 15,857 effect sizes published in 217 meta-analyses covering 13,632 experimental studies that took place between 1909 and 2020, across more than 150 countries. Our database covers interventions related to land-use change, land management, and also compiles studies on the impacts of climate change. We provide the explicit locations of the primary studies used in the meta-analyses, along with the type of interventions and outcomes analyzed, providing an opportunity to identify knowledge gaps. We also report possible trade-offs between SOC and, for example, crop productivity, or soil biological and chemical variables to explore the practical implementation of the investigated interventions. Finally, to provide guidance for future work, we also identify transparency and reproducibility issues of each of the 217 meta-analyses.

This database is relevant to advance the scientific and policy debate on the interest and possibility of implementing practical recommendations to increase SOC stocks, as proposed for instance by the 4p1000 Initiative, and thus contributing to climate change mitigation.

## Methods

### Data collection

**Fig. 1:**
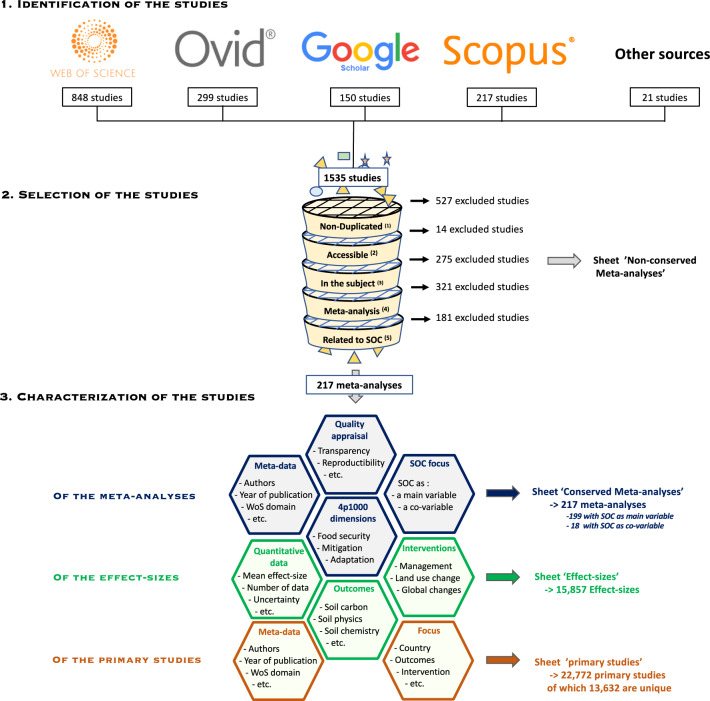
Methodological framework used to identify and characterize the data included in the database. Criteria for study selection are (1) duplicates are removed; (2) only studies published in English (non-English studies: n = 7) with available text (studies with non-available text: n = 7) are considered; (3) studies not dealing with SOC are excluded; (4) only meta-analyses are included; (5) the meta-analysis present at least one effect-size, i.e. a quantitative measure on SOC (or one effect-size described for different levels of SOC contents, i.e. SOC as a covariable). The hexagons represent the different characteristics analyzed in the meta-analyses or in the primary studies.

The literature search was performed on January 09, 2020 (Fig. 1). The following search equation was used: (“meta*analysis” OR “systematic review”) AND (“soil organic carbon” OR SOC OR “soil organic matter” OR SOM OR “soil carbon”) in the “topic words”, *i.e* titles, abstracts and keywords of the following databases:

- Web of Science, New York, USA, http://apps.webofknowledge.com, encompassing 12,000 journals and 160,000 conference proceedings.
- Scopus, USA, https://www.scopus.com/search. The Scopus database includes more than 41,000 referenced journals.
- OVID. Publisher, USA. https://www.ovid.com. The Ovid database includes more than 10,000 titles of scientific journals, books and proceedings (for Cab Abstracts on Ovid).
- Google Scholar. https://scholar.google.com/. Publisher: Google. It contains both, multidisciplinary peer reviewed and grey literature. We selected the first 150 search results, organized by relevance, as this engine is highly precise for the first pages of results displayed^23,24^.

Following the recommended golden standard of systematic review^25,26^, we used these different databases with various journal coverage to obtain an exhaustive literature search and to avoid potential bias. The search was supported by a librarian to further help to reduce the possibility of bias and improve the overall quality of the search strategy^27^.

No restriction on year of publication was applied. All climatic zones and countries were considered. Sensitivity was favored over specificity. Sensitivity implies that emphasis in the search procedure is put on collecting the largest selection of potentially relevant studies at the risk of also obtaining a high number of non-relevant studies (hence increasing the duration of the screening step). In addition to the database searches, a number of other potentially relevant meta-analyses were added by the authors of this study.

The literature search identified 1,535 studies (of which 1,008 were unique). These studies were compiled in a database and then screened to identify the relevant studies based on the following inclusion and exclusion criteria: (i) only studies published in English with available full text were considered; (ii) the study presents a quantitative, formal analysis of several previous empirical studies, *i.e.* a meta-analysis (we did not consider studies with vote-counting methods and narrative reviews were also excluded); (iii) the meta-analysis presents at least one effect-size, *i.e.* a quantitative measure of the magnitude, of a SOC variable, either as the main variable, or as a co-variable.

The studies were first screened based on the title and abstract, and if necessary, the whole manuscript was read. Each study was screened by two authors of the present article. The rejected studies were compiled in an exclusion sheet of the database, with reasons for exclusion. Finally, 217 meta-analyses fulfilled our inclusion/exclusion criteria, of which 18 with SOC as a co-variable.

### Characterization of the meta-analyses

Our database reports the meta-data (author names and affiliations, journal name, keywords, date of publication, and countries covered in the meta-analysis) for considered meta-analyses (Fig. 1). The transparency and reproducibility of each of the 217 meta-analyses was assessed based on criteria related to the literature search, data extraction, data analyses, and interpretations. These criteria are an adaptation of the ones proposed in several other studies covering various research fields^28–31^. When satisfied, a criterion was scored 1, and 0 otherwise. A global quality score was given by calculating the proportion of criteria met.

We also classified each meta-analysis for their scope, delineating whether they address “mitigation of climate change”, “adaptation to climate change” and “food security” dimensions. Definitions of the dimensions are based on the IPCC glossary^32^, and the objectives of the 4p1000 Initiative^15^ (https://www.4p1000.org/). Keywords related to each dimension (Table 1) were defined for the classification, that was manually performed by two different authors of this study. Title and abstract were screened, and the full text was studied if necessary. Consistency between reviewers was checked on a sample of 30 studies. The final database comprises respectively 199, 28 and 54 meta-analyses that analyse the mitigation, adaptation and food security dimensions.

**Table 1:** Keywords related to each 4 per 1000 dimension.

4 per 1000 dimension	Definition	Indicative keywords
Mitigation of climate change	A human intervention to reduce emissions or enhance the sinks of greenhouse gases^32^.	GHG emissions, GHG fluxes, CH_4_, N_2_O, CO_2_, carbon budget, carbon balance, soil respiration, anthropogenic emissions, cumulative emissions, depletion, loss, decarbonization, carbon sequestration, carbon stock, carbon storage, carbon sink, carbon pool, carbon source, accumulation rate, storage rate, stocks, tC, Mg, GtC, anthropogenic removals, carbon dioxide removal, GHG removal, negative emissions, uptake
Adaptation to climate change	In human systems, the process of adjustment to actual or expected climate and its effects, in order to moderate harm or exploit beneficial opportunities. In natural systems, the process of adjustment to actual climate and its effects; human intervention may facilitate adjustment to expected climate and its effects^32^.	yield, drought, dry spell, heavy rains, rainfall patterns, hydric stress, water stress, heat stress, thermal stress, flood, vulnerability, resilience, climatic variability, impact, effects
Food security	A situation that exists when all people, at all times, have physical, social and economic access to sufficient, safe and nutritious food that meets their dietary needs and food preferences for an active and healthy life^32^.	yield, food, diet, nourishment, human consumption, income

### Characterization of the effect-sizes

**Fig. 2:**
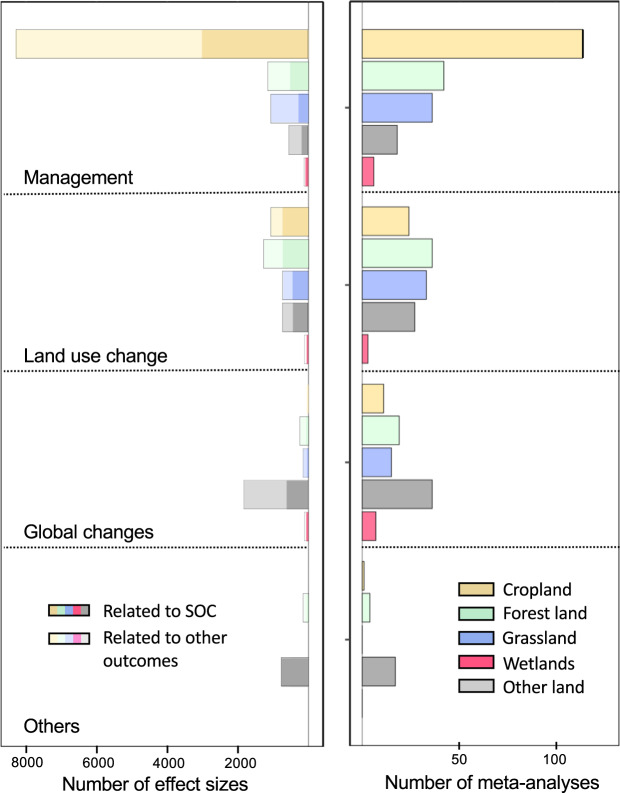
Number of effect sizes (left) and number of meta-analyses (right) available in the database pertype of intervention and land-use. The total number of effect sizes reported in the database is presented, together with the number of effect sizes on SOC (dark shaded) or on other outcomes (light shaded). For the land-use change intervention, both initial and final land-uses are considered in this plot. A meta-analysis can comprise different interventions or land-uses.

We extracted all the quantitative data related to the effects reported in the retrieved meta-analyses: effect size, indicator of dispersion (confidence interval, and/or standard deviation or quantiles), significance (P-value) and number of data on which the effects were calculated (Fig. 2). The database contains both the effect-sizes that quantified the direct effect of an intervention on SOC, and the effect-sizes that assessed the effect on other outcomes but with SOC as co-variable (indirect effects). Data were collected from tables or from figures using WebPlot Digitizer software (www.automeris.io/WebPlotDigitizer/). We also described the type of metric associated with each effect-size (*e.g.* mean difference, ratio, hedge’s *d*).

We did not extract data from meta-regressions, correlations and their associated characteristics because of the difficulty to synthetize this type of results across different studies. However, when subgroup analyses were performed (*e.g.,* by soil characteristics or climate zones - Table 2), we extracted the effects of the moderators in order to analyze and understand the variability of SOC values.

**Table 2:** Co-variables reported in the meta-analyses.

Co-variable	Percent of meta-analyses reporting the co-variable (%)
Biomes/climatic conditions	65.9
Detailed management practices	57.1
Soil depth	47.0
Soil types	47.0
Plant species	43.3
Experimental duration	42.9
Experimental methods/conditions	30.4

**Fig. 3:**
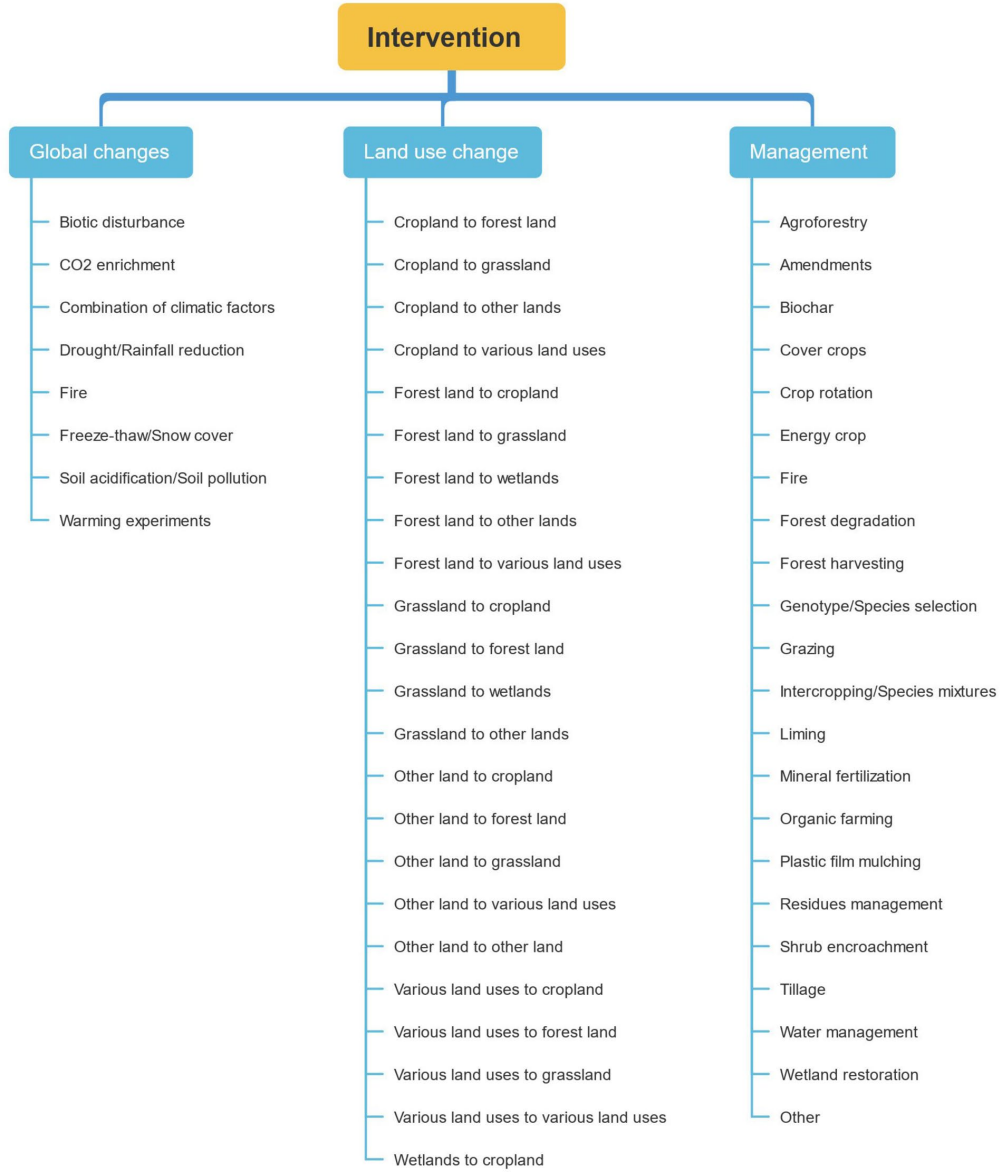
Categories used for the characterization of interventions for globalchanges, land-use changes and management.

The interventions related to the effect-sizes were grouped into main categories: land management, land-use change, and global changes (Figs. 2, 3). We considered land-use types as defined in the IPCC Guidelines for National Greenhouse Gas Inventories^33^: cropland, forestland, grassland, wetlands and other land. We considered land-use change as the conversion of one of the aforementioned land uses to another one. We considered land management as any intervention performed on any of the aforementioned land uses (*e.g.* forest harvesting, wetland restoration, mineral fertilization). We defined global changes as planetary-scale changes other than land-use change (*e.g.* climate change). The database presents broad categories of interventions, but also the more detailed interventions reported in each study (Fig. 3). The number of effect sizes and meta-analyses is largely dominated by studies of land management, particularly for cropland. This is followed by land-use change and global change type interventions, for which the distribution of land uses is more balanced.

**Fig. 4:**
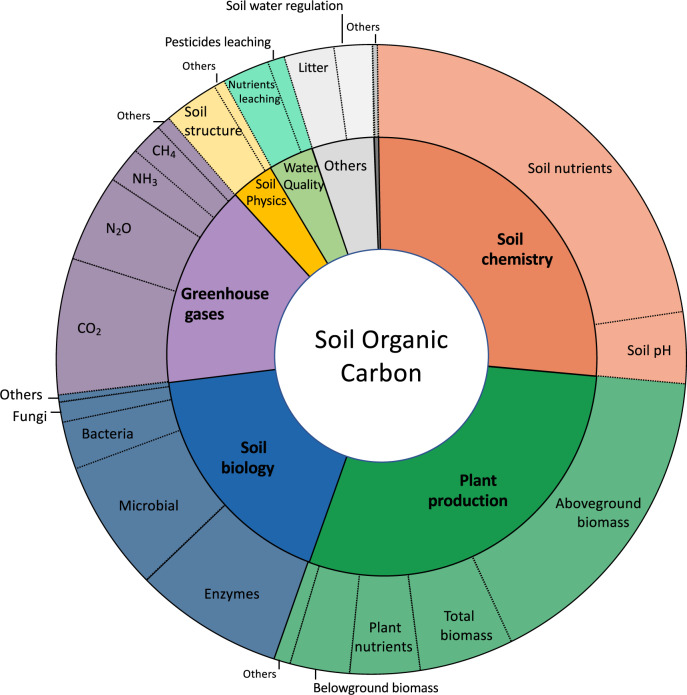
Main categories and subcategories of other effect-sizes retrieved in the 217 meta-analyses and studied concurrently with SOC. The areas are proportional to the number of effect-sizes in the database.

The outcomes other than those related to SOC were grouped into seven broad categories: soil chemistry, plant productivity, soil physics, soil biology, greenhouse gases, water quality and others. The first three categories represented nearly 20–30% of the reported effect sizes. Each category was further refined into 2 to 11 subcategories (Fig. 4). The soil nutrient and aboveground biomass subcategories alone represented nearly 15–20% of the effect sizes.

### Characterization of the primary studies used in the 217 meta-analyses

We retrieved all available references (primary studies) used by the 217 meta-analyses, by searching through the available list of references, supplementary materials and databases associated with each meta-analysis.

**Fig. 5:**
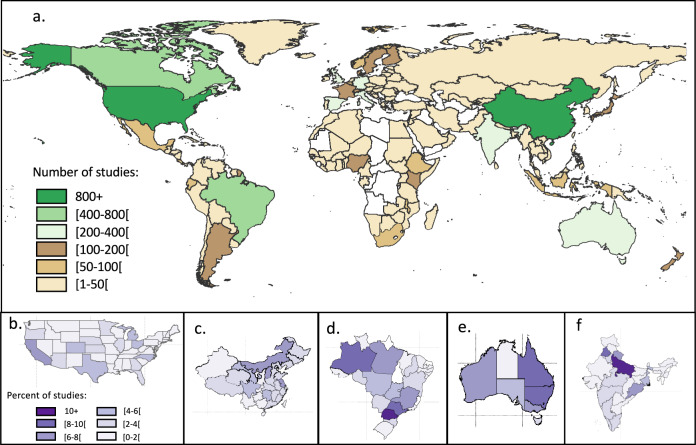
Geographical distribution of the 13,632 primary studies included in the 217 meta-analyses (**a**), with details of provincial/state distribution for (**b**) USA, (**c**) China, (**d**) Brazil, (**e**) Australia and (**f**) India.

The primary studies were characterized by their meta-data (*e.g.* DOI, authors, date of publication, journal). Based on the title and the abstract, if necessary, we also manually extracted the type of interventions and outcomes associated with the primary studies. The manual classification into the same intervention and outcome categories as described before, was facilitated by an automatic classification based on keywords (Supplementary Table 1). The final database comprises 13,632 unique primary studies (Fig. 5). 9,130 primary studies were used in several meta-analyses. The geographical distribution showed the highest number of these primary studies in the United States and China, followed by Brazil and Canada, and then Australia, India and some European countries (United Kingdom, Germany, Spain, and Italy). The regional distribution within the five countries with the largest number of studies showed great regional disparities. Africa was the least investigated continent; no primary studies were conducted in several African countries.

## Data Records

All data is available on the CIRAD repository, which can be accessed through the following link: 10.18167/DVN1/KKPLR8^34^. An excel file (version 16.16.27, 201012), with different sheets is provided, as described in Fig. 1:

- The sheet “readme” presents the contact information of the authors and the articles associated with the database.
- The sheet “retained meta-analyses”, includes the list of the 217 meta-analyses retrieved by our literature search and used for the analysis, their associated meta-data, and general characterization.
- The sheet “non-retained meta-analyses” includes the list of studies retrieved by the literature search but not used for the analysis, as well as the reasons for exclusion.
- The sheet “effect-sizes”, comprises all the effect-sizes extracted from the 217 meta-analyses. The effect-sizes are associated with a meta-analysis (with an ID), and characterized by the mean effect, indicators of dispersion (*e.g.* standard deviation, confidence interval) and the associated number of data used to calculate the effect-size.
- The sheet “primary_studies” includes the list of 22,772 (of which 13,632 are unique) primary studies collated from the 217 meta-analyses. The primary studies are associated with a meta-analysis (with an ID), and characterized by their meta-data, country, type of intervention and outcome analyzed.
- The sheet “header_names” describes the header of the four above-mentioned sheets.

## Technical Validation

The information extracted for each retained meta-analysis (general characterization, effect sizes and the associated primary studies) has been systematically double-checked by at least two different reviewers to reduce potential errors.

After the data extraction, we examined data quality using R software. We plotted the frequency distribution of the different variables, and returned to the original studies to verify any extreme values that were identified in this process. In this step we also randomly selected ~10% of extracted data to check for consistency by comparing them with the original data.

**Fig. 6:**
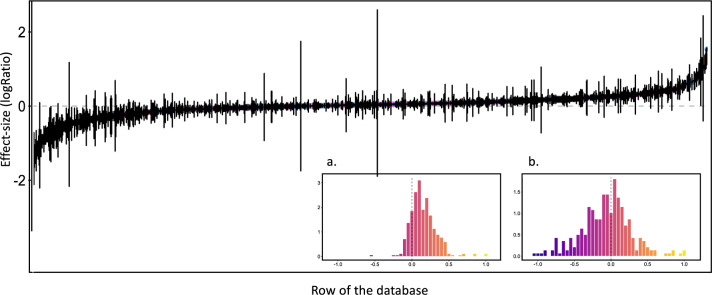
Values of the effect-sizes on SOC compiled in the database. The effect-sizes are presented as log ratios, with the bars representing the lower and upper confidence intervals. Subset **a** and **b** present histograms of the mean values of the effect-sizes for all drivers in cropland (**a**), and for land-use change (**b**).

The list of included and excluded studies were further checked by creating a PRISMA diagram (Fig. 1). The quality criteria of the meta-analyses were checked and analyzed in detail in^35^. The ‘effect-size’ sheet was examined by ii) plotting the number of data available per outcome, per sub-category of outcome (Fig. 4), per land-use type (Fig. 2), and per intervention (Figs. 2, 3, in^35^), iii) plotting the value of the effect sizes and their associated standard errors globally (Fig. 6) and for each combination of intervention and outcome. The information of the ‘primary studies’ sheet was examined by i) mapping the number of studies by country, or by region for the main countries (Figs. 3, 5 in^35^), and ii) analyzing the number of common studies between the meta-analyses (Fig. 4 in^35^).

## Usage Notes

To our knowledge, this database constitutes the most comprehensive database based on published meta-analyses for analyzing the potential of various interventions for storing SOC. The database is useful for scientists and decision makers in defining relevant climate change policies to limit global warming based on robust experimental evidence. It can be used to analyze the expected efficiency and associated uncertainty of proposed solutions to store C in soils. As we systematically extracted all available data on SOC, our database can also inform about possible knowledge gaps, and can be used to guide future research agendas.

Since we extracted all variables and moderators presented in the meta-analyses, the database also provides a good basis for analyzing trade-offs between SOC and other related variables. Numerous studies have indeed demonstrated that trade-offs can limit the efficiency of an intervention in mitigating climate change, as for example shown by the trade-off between SOC storage and N_2_O emissions^36^. The numerous non-SOC effect-sizes comprised in our database can be a good starting point for systematic reviews on other outcomes (*e.g.* impacts of agricultural practices on GHGs, or on soil quality).

Importantly, our database can be easily updated using new data produced by the increasing number of meta-analyses published on this topic. We welcome anyone interested to share data or studies not included in this meta-database and send them to the corresponding author. We will add the new observations and update our database regularly with the latest experimental data.

The database is currently being integrated into a web platform providing user-friendly visualisation of the data and results of soil carbon studies (http://www.review4c.net).

## Supplementary information

Table S1

## Data Availability

The codes that were used to make the graphs presented in this paper are available at the following address: https://github.com/dbeillouin/Data_Paper_SOC#readme.
