# Supplementary material for: A global database of land management, land-use change and climate change effects on soil organic carbon
Source: Sci Data. 2022 May 24;9:228. doi: 10.1038/s41597-022-01318-1 (PMC9130507; doi:10.1038/s41597-022-01318-1)
Supplement: Supplementary file 1 — Table S1 [file 41597_2022_1318_MOESM1_ESM.docx]

**Supplementary table 1.** Keywords related to each intervention and outcome categories.

| **Category** | **Keywords** |
| --- | --- |
| *Plant_Production* | crop uptake, plant cover, plant community, plant communities, root, roots, stand structure, stand density, forest structure, radiation interception, leaf-area, biomass, forage, production, productivity, yield, yields, dry matter, land equivalent ratio, leaf area index, growth, performance, virus, striga, aphids, aphid, herbivorous, herbivore, parasitism, damage, pest, disease, rust, mildew, parasitoid, predators, natural enemies, weed, stemborers, take-all, N status, nitrogen contribution, energy use, nitrogen uptake, nitrogen-15, fix nitrogen, ^15^N, input use, nitrogen use, nitrogen utilization, N fixed, ndfa, nitrogen fixation, nodulation, fertilizer use efficiency |
| *soil_chemistry* | nitrogen balance, nitrogen balance, n availability, nitrogen storage, nitrogen stocks, nitrogen pool, nitrogen budgets, nitrogen stocks, soil-pH, soil salinity, fate of N, nitrogen availability, recovery of fertilizer, fate of N-15, fate of (15) N, nitrification, N content, N fluxes, chemical-properties, nitrogen content, soil acidification, soil acidity, soil N and P, N_2_ fixation, litter decomposition, fate of fertilizer, chemical-properties, amino nitrogen, soil C and N, biochemical activities, nitrogen fractions, soil chemistry, chemical composition, P chemistry, potassium, soil stoichiometry, nitrogen availability, fates of N-15, heavy metals, fate of (15) N, fate of nitrogen, selenium, nitrogen retention, soil phosphorus, phosphorus, nitrogen-fixation, soil chemical, N cycling, N dynamics, N cycle, P availability, nutrient, nutrients, nitrogen source, nitrogen cycling, mineralization, soil nitrogen, soil potassium, calcium, CEC, nitrogen dynamics, magnesium, chemical properties |
| *Soil_biology* | enzyme activity, enzyme activities, fungi, fungal, biological indicators, respiration, soil microbial, microorganism, microbial, microbiological, heterotrophic respiration, soil biology, soil enzymatic, soil enzyme, soil nematofauna, macrofaunal communities, soil biota, soil enzyme activity, soil biotic processes, soil biochemical, soil biological, soil fauna, biological properties, arbuscula, collembola, bacteria, mycorrhizal, nematode, carabid, earthworms, earthworm, diversity, bird, beetle, butterfly, nematodes, invertebrates, invertebrate, biodiversity, arthropods, arthropod, biological control, colonization, soil respiration, mycorrhizal |
| *Assoc_biodiv/ Others* | plant community responses, butterflies, insect, insects, bees, bumblebees, bee, mammal, bats, pollination, spider, pollinator, pollinators |
| *GHG* | carbon dioxide, carbon dioxide fluxes, carbon exchange, carbon budget, carbon dioxide exchange, nitrous oxide, nitrous oxide emissions, nitrous oxide fluxes, warming potential, NH_4_, NH_3_, CO_2_, CH_4_, N_2_O, volatilization, emission, denitrification, methane, greenhouse |
| *Soil physics* | aggregate size, soil attributes, soil response, soil parameters, soil aggregate, soil property, soil restoration, physical-properties, soil degradation, soil health, soil quality, fertility, soil indicators, physical characteristics, soil compaction, soil compressibility, aggregate stability, soil processes, soil aggregation, soil pore, soil characteristics, physical properties, porosity, soil physical, compaction, bulk density, soil stability, soil resistance, soil structure, soil properties |
| *Water_quality* | Soil erosion, erosion, leaching, nitrate, soil loss, sediment, herbicides, pesticides, removal, pollution, nitrogen loss, losses |
| *carbon* | dynamics of carbon, carbon sink, storage of C, carbon stores, carbon and nitrogen sequestration, stock of carbon, carbon fractions, organic-carbon, C dynamics, carbon allocation, carbon turnover, carbon balance, carbon pool, carbon pools, carbon and nitrogen storage, carbon and nitrogen stocks, carbon and nitrogen pool, carbon and nitrogen budgets, N carbon accumulation, cycling of carbon, carbon pools, accumulation of carbon, soil labile carbon, C and N content, carbon storage, soil carbon, organic matter, organic fractions, C quantities, C sink, C stocks, organic C, C organic fractions, SOC, soil organic-carbon, soil organic carbon, C pools, carbon sequestration, soil organic, C storage, carbon stock, ecosystem carbon |
| *Land-use Change* | grassland degradation, deforestation, restoration, land abandonment, land-use, land-use, conversion, vegetation restoration, forest conversion, land-use change, forest-to-pasture conversion, land-uses, land management, reforestation, land-use intensification, rehabilitated mangrove, land-use type, conversion of cropland, afforestation, forest restoration, encroachment |
| *Management* | nutrient additions, fertilized, farming, cultivation, nitrogen fertilizers, organic agriculture, managed, clearing, cover-crop, cover crop, yield, harvesting, lime, fertilization, residue incorporation, nitrogen amendement, nitrogen inputs, crop productivity, cropping system, organic farming, plantation, plantations, maize, no-till, trifolium, biosolid, biosolids, cover crops, farming systems, nitrogen fertilisation, thinning, crop residue, nitrogen fertilization, phosphorus addition, alley farming, hedgerow, potato, paddy, amended, nutrient addition, sugar beet, norway spruce, cropping sequences, rice, compost, tree coppicing, forest clearing, corn, liming, rotational, management, conservation agriculture, nitrogen addition, biochar, crop-residue, upland, cultivated, alfafa, wheat, forest management, intercropping, alley cropping, logging, grazing, cropping systems, agroforestry, long-term fertilization, tillage, crop residues, manure, amendements, conservation tillage, crop rotation, fertilizer management, forest management, alpine grassland, grassland management |
| *Global_Changes* | pollution, wildfires, carbon dioxide increases, burning, wildfire, contamination, CO_2_ enrichment, elevated CO_2_, invasive plant, freezing, [CO_2_], fire, burned, invasion, invasive, snow, rainfall variability, atmospheric CO_2_, increased CO_2_, climate change, climate change, disturbance, CO_2_-induced, elevated-CO_2_, elevated carbon dioxide, burning, CO_2_ exposure, elevated [CO_2_], freeze-thaw, elevated CO_2_, atmospheric CO_2_, precipitation, fire, warming, drought, snow |
